# Practical Tips for Facilitating Research

**DOI:** 10.5195/jmla.2017.114

**Published:** 2017-01

**Authors:** Paul M. Blobaum

Published in March 2016, this book is the first in Facet Publishing’s planned Practical Tips for Library and Information Professionals Series, edited by Helen Blanchett. The series is envisioned as a set of practical guides for busy professionals, grounded in theory but full of practical advice and best practices contributed by practitioners for “information professionals looking for new ideas and inspiration.”

Moira Bent is an academic librarian and National Teaching Fellow at Newcastle University, Newcastle upon Tyne, England, United Kingdom, where she assumed the role of “faculty research support.” Bent and her colleagues conducted research on their campus that sought to identify what support was needed and could be provided by the librarian liaison program, resulting in the publication of research in ResIN: Research Information at Newcastle, predecessor of the library’s impressive and informative research support web page.

In the introduction (section 1), Bent explains that the book is not meant to be read cover-to-cover but to be used for “dipping in and out.” Bent acknowledges the book holds a bias toward the UK perspective, but this reviewer finds that, as Bent hoped, most of the contributions are applicable inter nationally and in multi-type libraries. In section 2, Bent summarizes the eight subsequent sections: “Landscapes and Models” (section 3), “Structures and Strategies” (section 4), “Places and Spaces” (section 5), “Library Staff Roles” (section 6), “Collections” (section 7), “Specific Interventions in the Research Process or Lifecycle” (section 8), “Teaching Approaches” (section 9), and “Information Literacy Skills Workshops and Programmes” (section 10).

Each section contains five to twenty-four tips, with examples from practice, points to think about, and ample references for in-depth reading. For example, 5.3 “Developing Research Zones” gives a brief overview of having designated space in the library for users who are working on research projects and explores the “Research Commons” concept. Examples are given from several university libraries under the heading “Examples from Practice.” Under “To Think About,” three bullet points highlight the issues of access policy, benefits of defining a space and impact on the wider research community, and consideration of whether the space should be inside the library or closer to the researchers. These innovative features distinguish the book from the only similar title on the subject of librarians and support for research, *Dynamic Research Support for Academic Libraries,* edited by Starr Hoffman, also published in 2016 (Chicago, IL: Neal-Schuman; ISBN: 978-0-8389-1469-4).

Facet Publishing, publisher of the UK Chartered Institute of Library and Information Professionals (CILIP), and Neal-Schuman, publishing arm of the American Library Association, have a close partnership. The Hoffman title is published by Facet in the United Kingdom; one wonders how similar titles with similar pricing and the same target audience would appear on the market in the same month and year.

Bent’s book leaves no topic unturned and is full of ideas to put into practice immediately. The complete research process of planning to publication to data management of scholarly communication is covered thoroughly, including crash courses on teaching, writing, and submitting manuscripts; how to avoid predatory publishers; and open access. The publisher list price may appear steep, but paperback copies can be purchased for much more reasonable prices, depending on the vendor. This book is recommended for academic librarians and health sciences library collections that support researchers. Researchers and students will also benefit from adding this book to their personal collections.

